# Cellular imaging using emission-tuneable conjugated polymer nanoparticles†

**DOI:** 10.1039/c9ra07983a

**Published:** 2019-11-20

**Authors:** Struan Bourke, Yurema Teijeiro Gonzalez, Federico Donà, Maryna Panamarova, Klaus Suhling, Ulrike Eggert, Lea Ann Dailey, Peter Zammit, Mark A. Green

**Affiliations:** Department of Physics, King's College London London WC2R 2LS UK mark.a.green@kcl.ac.uk; Randall Division of Cell and Molecular Biophysics, Faculty of Life Sciences and Medicine, New Hunt's House, King's College London Guy's Campus London SE1 1UL UK; Institute of Pharmacy, Martin-Luther University Halle-Wittenberg Wolfgang-Langenbeck-Strasse 4 06120 Halle (Saale) Germany

## Abstract

New materials that exhibit tuneable optical properties, notable emission across the visible spectrum, are of immense interest to biologists as they present a broad palette of colours from a single imaging agent that can be utilised in biological detection. Such a flexible system, when combined with the advantages of using conjugated polymer nanoparticles in cell imaging results in a widely useful medical diagnostic system. Here, we describe tuneable emission observed through oxidation of a conjugated polymer followed by the formation of nanoparticles and their subsequent use in cell imaging.

## Abstract 2

In this report, we report the synths of colour tuneable conjugated polymer nanoparticles.

## Graphical abstract

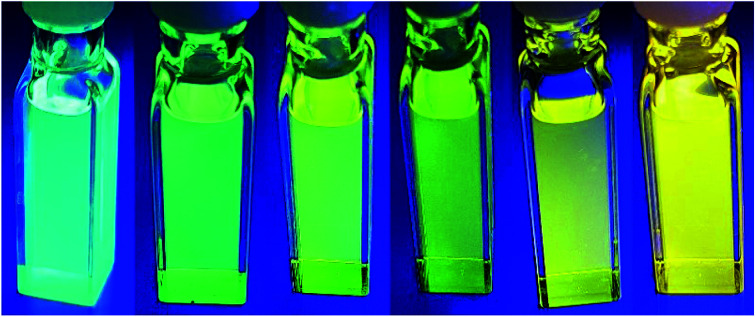

## Introduction

Nanomaterials have provided a new family of diagnostic tools for cellular imaging, with quantum dots (QDs) being, to date, the most successful. One of the defining features of QDs is their tuneable optical properties with respect to spatial dimensions.^1^ Although slight shifts in emission are routinely observed for numerous materials under differing conditions, they are generally insignificant with respect to spectral range and colour purity. Whilst QDs are notable for their tuneable emission, the heavy metals of which they are composed makes for limited use in clinical applications.^2–4^ Conjugated polymer nanoparticles (CPNs), also referred to as polymer dots (P-Dots) and semiconductor polymer nanoparticles (SPNs), have distinct advantages over traditional luminescent nanomaterials and have shown great promise in biological imaging due to their notable optical properties,^5–12^ including their bright emission, large absorption coefficients, enhanced stability and biologically inertness (circumventing the issue of heavy metal toxicity in QDs).^9,13^ The particles are however limited by the range of available colours, meaning that multispectral imaging requires different sets of conjugated polymers which have separate chemical structures which affects optical and physical characterisation when these structures are entrapped in micelles. One advantage of QDs is the inherent tunability due to size quantisation effects,^14^ allowing a wide spectral region to be accessed using a small number of materials. Whilst CPNs do not exhibit classic size quantisation effects due to carrier confinement, we have utilised oxidation to controllably tune the emission of a single polymer species (poly[2-methoxy-5-(2′-ethylhexyloxy)-*p*-phenylenevinylene], MEH-PPV) though the visible spectrum. The method employed is inexpensive and the resulting conjugated polymers remain brightly fluorescent whilst exhibiting colours from blue to red dependent on the amount of oxidising agent used.^15–18^ The oxidised polymers were further processed into nanoparticles using the nanoprecipitation method with poly(styrene-*co*-maleic anhydride) (PSMA) as a surface species, encapsulating the CP due to their inherent hydrophobicity.^16^ At the same time, we were interested in small and highly stable CPNs, and thus looked at the self-assembling Pluronic® F127 to form stable micelles containing the emitting polymer. We further encapsulated magnetic nanoparticles within the CPNs, potentially adding another imaging modality whilst making separation simpler. Biological imaging of the CPNs was demonstrated using two mammalian cell lines (HeLa and HCE). To determine the viability of these CPNs, their cytotoxicity was evaluated by life/dead fluorescence stains in HeLa and HCE cells and an ATP luminescence assay in HEK cells. The potential of these nanoparticles as fluorescent probes is shown *via* the uptake of the particles by the different cell lines and subsequent imaging with a confocal scanning laser microscope.

## Results and discussion

**Fig. 1:**
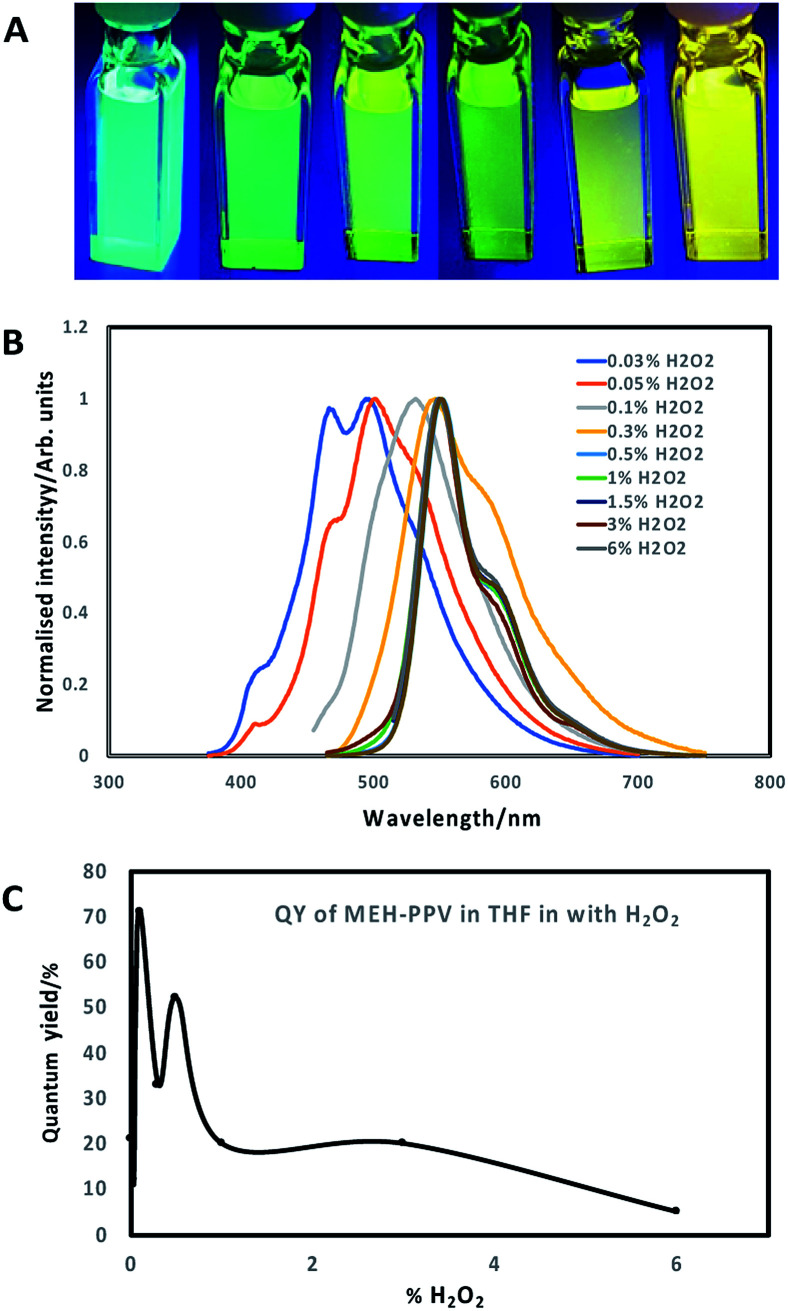
(A) Images of oxidised MEH-PPV stock solution in THF; (B) normalised emission spectra of oxidised MEH-PPV in THF following 7 days incubation with H_2_O_2_ at different concentrations (excitation 365 nm); (C) QY% of oxidised MEH-PPV in THF.

Tetrahydrofuran (THF) was utilised as a water-miscible aprotic solvent that is often used in polymer nanoparticle preparation. However, one issue with using THF is its degradation into peroxides, which are potent oxidising agents, in the presence of air. Normally, THF contains butylated hydroxytoluene that prevents oxidation from occurring, however THF can be supplied without an inhibitor.^19^ MEH-PPV was dissolved in THF (without butylated hydroxytoluene) and then mixed with a dilute solution of hydrogen peroxide (H_2_O_2_) in the dark at room temperature for several days. ESI (Fig. S1a and b†) show the absorption and photoluminescence spectra of the resulting solution at day 0, retrospectively highlighting no change in emission, although addition of 0.03% H_2_O_2_ resulted in an immediate blue shift in the absorption spectra maxima of *ca.* 50 nm. A gradual blue-shift in the absorption and emission spectra of the solutions (Fig. 1A and B; ESI Fig. S1c and d†), was observed after 7 days, with a shift-dependence on H_2_O_2_ concentration. Solutions with H_2_O_2_ concentration of 0.3% and lower showed a blue shift in the absorption spectra from *ca.* 500 nm to below 400 nm. Similarly, the emission peaks shifted towards the blue end of the visible spectrum (from *ca.* 575 nm maxima to *ca.* 500 nm maxima) upon addition of H_2_O_2_ solutions of 0.1% concentration and lower. Surprisingly, higher percentages of H_2_O_2_ did not significantly change the photoluminescence or absorption spectra of the MEH-PPV. Values of maximum wavelengths for the spectra at different concentrations of H_2_O_2_ is shown in ESI, Table S1.†

The oxidation and shift in emission was likely due to a combination of both saturation of the bonds at the vinylene linkages and epoxidation of ethylene moieties over time to form epoxide rings, which increased the number of conjugation breaks resulting in the noted blue shift.^15,16,18,20^ Another feature that was noted was that the quantum yields of the oxidised MEH-PPV increased significantly. Pristine MEH-PPV in THF has a QY of 21%,^15^ whereas the oxidised MEH-PPV had a maximum quantum yield of 71% (0.1% H_2_O_2_, 7 days) (Fig. 1C). Values of QY for the oxidised MEH-PPV at different concentrations of H_2_O_2_ is shown in ESI, Table S1.†

**Fig. 2:**
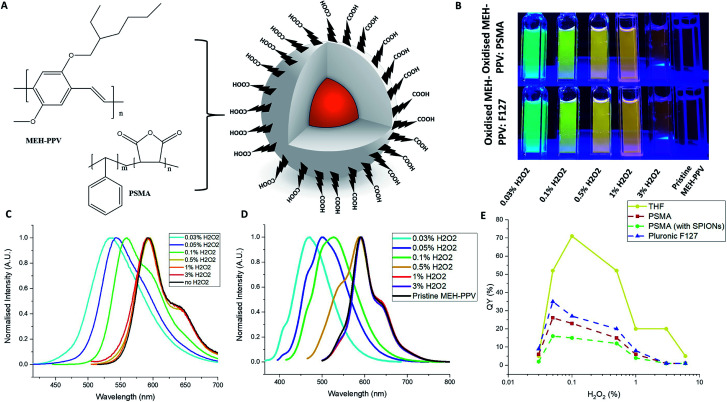
(A) Schematic of oxidised MEH-PPV:PMSA nanoparticles. (B) Images of oxidised MEH-PPV incorporated in PMSA nanoparticles or Pluronic F127 micelles. Normalised photoluminescence spectra of oxidised MEH-PPV:PSMA nanoparticles (C) and MEH-PPV:Pluronic F127 nanoparticles (D) (excitation either at 365 or 500 nm). (E) QY% of oxidised MEH-PPV nanoparticles compared to THF solutions.

The oxidised conjugated polymer dispersions (with/without iron oxide nanoparticles) were then used to prepare conjugated polymer nanoparticles as described in the ESI,† with either PSMA or Pluronic® F127 as shown in Fig. 2A. The nanoparticles were stable and optically clear for at least a month without aggregation (Fig. 2B). Fig. 2D presents the emission spectra of the different nanoparticles *vs.* pristine MEH-PPV (no H_2_O_2_, black line). Absorption spectra can be found in the ESI Fig. S2 and S3.† The oxidised MEH-PPV CPNs exhibited a shift in emission compared to oxidised MEH-PPV in solvent, which is a noted phenomenon in conjugated polymer aggregates and thin films^21–23^ due to the polymers interacting closely with each other causing delocalisation of the π-electrons, resulting in increased inter-chain aggregated states,^24^ presenting as a broadening in the absorption spectra, a red shift in the photoluminescence and a reduction in QY% (Fig. 2E). Numerous groups have reported similar observations based on the photo-physical behaviour of water-dispersed conjugated polymer nanoparticles.^5,25,26^ The F127 block copolymer encapsulated the oxidised MEH-PPV as a micelle (with the poly(propylene oxide) (PPO) blocks as the core and poly(ethylene oxide) (PEO) blocks as the shell). Both the absorption and photoluminescence spectra showed a slight blue shift compared to the oxidised MEH-PPV encapsulated in PSMA, with absorption maxima between *ca.* 350 nm and 525 nm, and associated emission maxima between *ca.* 450 nm and 600 nm (ESI Fig. S2†).

Overall, all the QYs of the NPs encapsulated in either PSMA or F127 showed a decrease when compared to the free oxidised polymer in organic solution although this varied widely with regards to the amount of hydrogen peroxide used in the reaction (Fig. 2E).

The decreased emission of the CPNs relative to the oxidised polymer form is due to defects in the polymers entrapped within the particles. The nanoparticles (encapsulated by either PSMA or F127) of the MEH-PPV oxidised by 3% H_2_O_2_ showed a decrease in quantum yield from 20% (in THF) to 1%. The nanoparticles encapsulated by F127 had slightly higher quantum yields, compared to those nanoparticles encapsulated by PSMA. The maximum QY of the oxidised MEH-PPV nanoparticles was 35% (MEH-PPV oxidised by 0.05% H_2_O_2_ and encapsulated in F127), notably higher than the unoxidized polymer in THF, which has been noted elsewhere (Fig. 2E).^15^

Values of absorption and photoluminescence spectra and QYs for the oxidised MEH-PPV nanoparticles are shown in ESI, Table S2.†

**Fig. 3:**
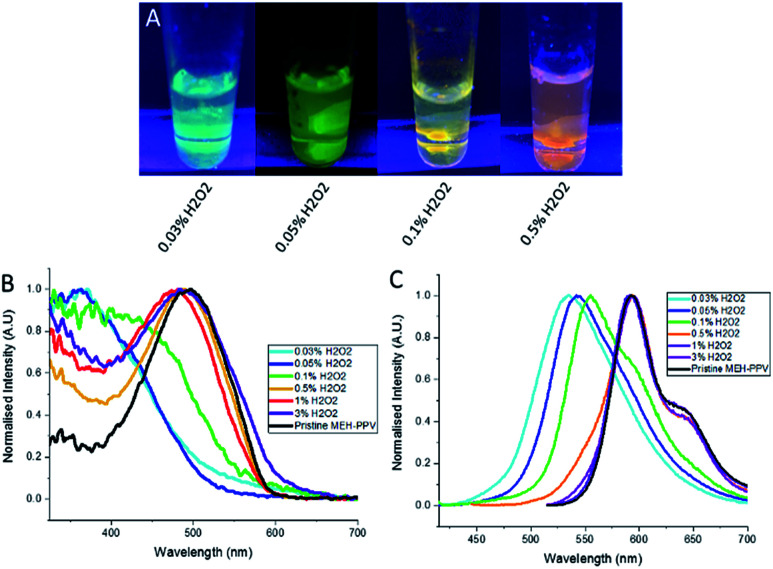
(A) Photograph shows emission colours of the different oxidised MEH-PPV:PSMA NPs with SPIONs at 10 μg mL^−1^ against a magnet (excitation at 365 nm). (B) Normalised absorption spectra of oxidised MEH-PPV nanoparticles (+SPIONs), and (C) normalised photoluminescence spectra of oxidised MEH-PPV nanoparticles (+SPIONs). Excitation was either at 365 or 500 nm.

We also prepared CPNs which incorporated magnetic nanoparticles. By adding superparamagnetic iron oxide nanoparticles (SPIONs) at the same time as the oxidised MEH-PPV and PSMA, the SPIONs were encapsulated inside the PSMA due to their hydrophobic nature. The resulting particles maintained their tuneable emissive properties as evidenced by the emission and absorption spectra whilst responding to an external bar magnet (Fig. 3).

The size of the particles was determined using dynamic light scattering (DLS). The majority of the NPs lacking SPIONs had a hydrodynamic diameter of between 60 and 70 nm, though the NPs made from 3% H_2_O_2_ stocks were *ca.* 150 nm (ESI Fig. S4†). The NPs made from the 0.03%, 0.05%, 0.5% and 0.1% H_2_O_2_ stock solution had relatively narrow size distributions with low polydispersity indexes (PDIs) of between 0.1 and 0.2 compared to the PDI of the particles made from 1% and 3% H_2_O_2_ stocks, which had PDI values of 0.3. Nanoparticles that contained SPIONs were slightly larger, ranging in size from *ca.* 60 nm to 180 nm with a PDI of *ca.* 0.3 suggesting polydisperse samples. A similar trend in size and PDI was noted with the oxidised MEH-PPV encapsulated with Pluronic® F127, with an average size of around 60 nm and PDI of 0.3 (ESI, Fig. S4,† blue bars). Values of size and PDI for the oxidised MEH-PPV nanoparticles are shown in ESI, Table S2.†

**Fig. 4:**
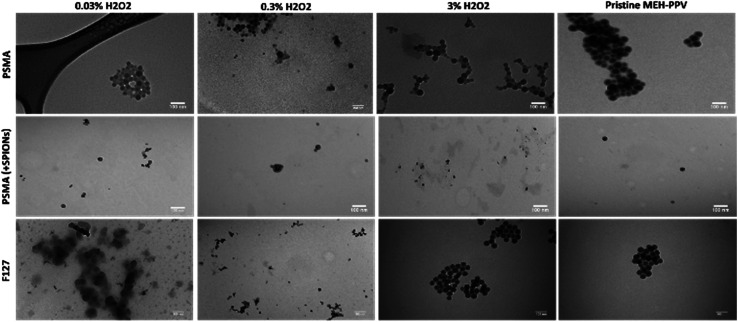
TEM images of the oxidised MEH-PPV, Taken at 100× resolution (scale bar = 100 nm). Top line is oxidised MEH-PPV:PSMA NPs, middle line is oxidised MEH-PPV:PSMA:SPIONs, and bottom line is oxidised MEH-PPV:F127.

Transmission electron microscopy (TEM) revealed approximately spherical colloidal nanostructures, as shown in Fig. 4 which aggregated when dried on TEM grids. As most of the constituents were composed mostly of organic polymers, there was little contrast whilst SPIONs could be observed as black dots encapsulated inside the particles. The oxidised MEH-PPV:PSMA nanoparticles (with and without SPIONs) showed a hydrodynamic diameter between 40 and 50 nm, with the SPIONs (black dots) observed as between 5 and 10 nm. The oxidised MEH-PPV:F127 nanoparticles showed an average size of 50 nm. The difference between the sizes of the particles observed by TEM *vs.* the DLS measurements was attributed to the hydrated size of the particles in water. The zeta potential was also measured using the Malvern Zetasizer in deionised H_2_O at 25 °C, with PSMA-coated NPs having a zeta potential of −30 mV, while Pluronic® F127 CPNs exhibited a more neutral zeta potential of −10 mV.

**Fig. 5:**
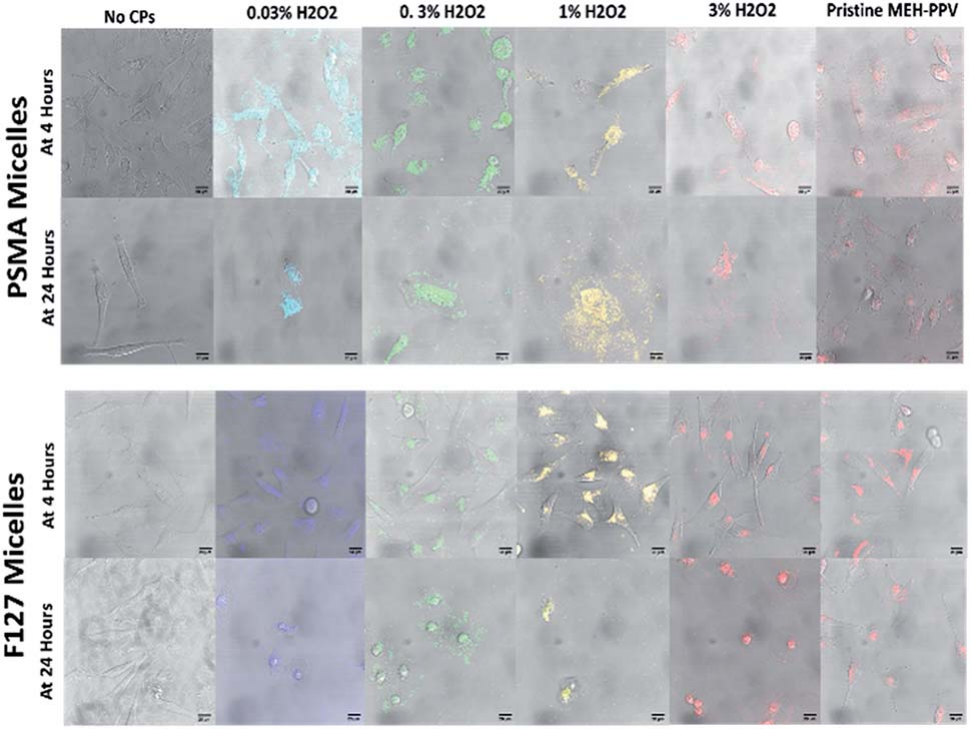
HeLa cells untreated or treated with oxidised MEH-PPV:PSMA NPs of different colours after 1 hour or 4 hours at 63× magnification with a total solid concentration of nanoparticle at 10 μg mL^−1^. Fluorescence was detected as follows: for 0.03% H_2_O_2_ oxidised MEH-PPV:PSMA NPs between 475 and 515 nm; 0.3% H_2_O_2_ oxidised MEH-PPV:PSMA NPs between 525 and 575 nm; 1% H_2_O_2_ oxidised MEH-PPV:PSMA NPs between 550 and 600 nm; 3% H_2_O_2_ oxidised MEH-PPV:PSMA NPs between 575 and 650 nm; and pristine MEH-PPV:PSMA NPs between 575 and 650 nm. Scale bars = 10 μm.

To investigate their potential use in biological imaging, the cellular uptake of the oxidised MEH-PPV NPs (both with PSMA and F127) by HeLa cells were evaluated by an inverted confocal microscope. The CPNs were initially incubated with HeLa cells (gifted and used in the Carlton lab and validated by STR profiling from Eurofin MWG)^27^ at a low concentration (5 μg mL^−1^ total solid) and imaged at 4 and 24 hours. After incubation, the cells were washed with PBS before being fixed in 10% formalin. It was noted that after 4 hours, the NPs appeared to be taken up by the HeLa Cells (Fig. 5) However, after 24 hours, the cells appeared to be undergoing apoptosis and after 24 hours, the cells were no longer viable.

This was repeated with a lower concentration of NP (1 μg mL^−1^ total solid), but after 24 hours the plate showed the cells had undergone apoptosis with 90% cell death in the wells treated with oxidised MEH-PPV:PSMA NPs. Both pristine MEH-PPV:PSMA NPs and the negative control showed less than 1% cell death.

**Fig. 6:**
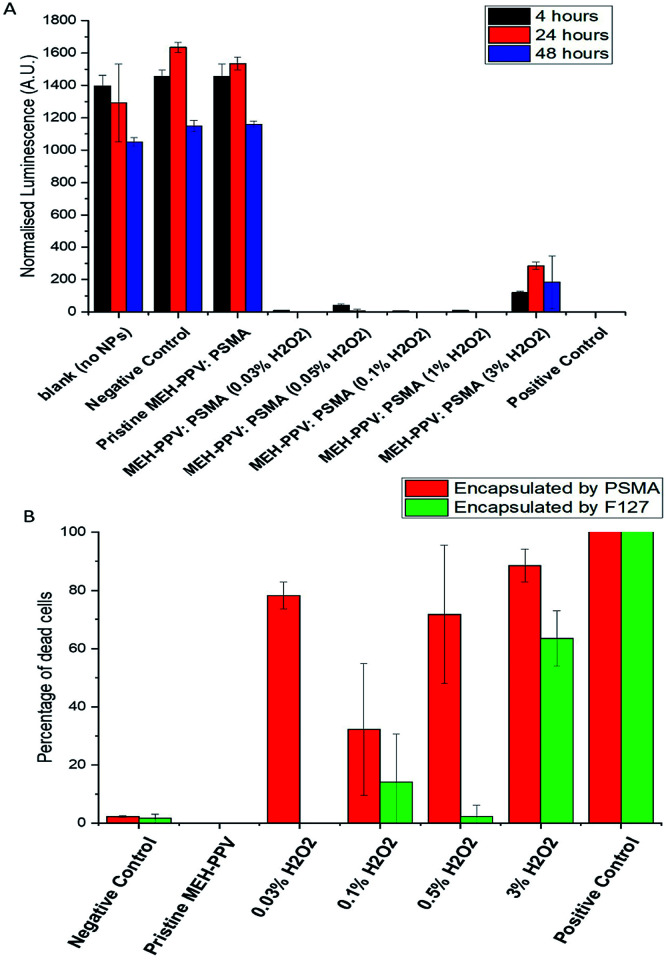
(A) Normalised oxyluciferin luminescence (a.u.) of different oxidised MEH-PPV:PSMA systems incubated for 4, 24 and 48 with HEK cells compared to wells with medium only (blank) and negative controls (PMSA nanoparticles without MEH-PPV). All values were normalised against blank control wells with no nanoparticles (*n* = 3). The positive control was PSMA NPs that been mixed with H_2_O_2_ (at 1%)s. (B) Live/dead cell imaging in HCE cells following 24 incubation with oxidised MEH-PPV PMSA and F127 CPNs. Negative controls consisted of PMSA and Pluronic® F127 without MEH-PPV. Positive controls consisted of was PSMA or F127 NPs that been mixed with H_2_O_2_ (at 1%) is a positive control. Quantification of NucGreen positive cells are presented as mean ± SEM (*n* = 3), >50 cells were scored per experiment.

The cytotoxicity was evaluated by measuring the *in vitro* viability of human embryonic kidney cells 293 (HEK293) (gifted by Maryna Panamarova from the Zammit Group, Randall Division of Cellular and Molecular Biophysics, King's College London) using CellTiter-Glo® Luminescent Cell Viability Assay (Promega, UK) which determined the number of viable cells in culture based on quantitation of the ATP present, to give an indication of metabolically active cells. Fig. 6A shows normalised luminescence from the assays after 4, 24 and 48 hours of exposure to CPNs. Values were normalised against the control medium containing no particles and showed that all oxidised MEH-PPV CPNs capped with PSMA were toxic after 4 hours. Subsequently, the cytoxicity of PSMA was compared to that of Pluronic® F127 CPNs in a non-cancer cell line, human corneal epithelial cells (HCEs) (gifted from Min S. Chang, Vanderbilt University, Nashville, Tennessee).^28^

The cells were incubated for 24 hours with the CPNs, at 5 μg mL^−1^, and then stained with a live/dead cell viability imaging kit (Thermofisher Scientific) to visualise the number of live *vs.* dead cells during incubation. This assay indicated that the cytotoxicity was related to the amount of peroxide used to produce the oxidised MEH-PPV (6b). Furthermore, it appeared that the encapsulation of the oxidised MEH-PPV in Pluronic® F127 reduced the cytotoxicity of the system. CPNs made with pristine MEH-PPV showed no evidence of cytotoxicity and were similar to negative controls.

As the PSMA-only NPs and the pristine MEH-PPV CPNs did not appear to have an adverse effect on the cells, we suggest that any cytotoxic effect was due to H_2_O_2_ remaining in the nanoparticle, although this has yet to be confirmed. However, Bellacanzone *et al.* and Feng *et al.*^29^ highlighted similar systems generated free radicals or reactive oxygen species. The slow generation of free radicals upon photoexcitation of a luminescent material presents a possible theranostic application, especially as this appears controllable.

## Conclusions

In conclusion, the emission of the conjugated polymer MEH-PPV was tuned from 490 to 550 nm with an increase in emission quantum yield through oxidation. By entrapping the emitting polymer within the self-assembling amphiphilic polymer PSMA, it was possible to make small, monodispersed, stable nanoparticles that had red-shifted emission with quantum yields from 2 to 18%. These nanoparticles could be magnetised through the addition of SPIONs, which exhibited similar emissive properties, but showed an increased size. Upon incubation with HeLa cells, bright fluorescence was observed within the cells after 1 hour, however after 4 hours the cells exhibited cytotoxic effects, which was also observed in HCE cells however, the cytotoxicity appeared to be reduced by using F127 as a capping agent. The ability to tune emission and cell toxicity offers potential theranostic applications, suggesting that conjugated polymer nanoparticles are not simple imaging agents, but offer a plethora of biological applications.

## Conflicts of interest

There are no conflicts to declare.

## Supplementary Material

RA-009-C9RA07983A-s001
