# Supplementary material for: Cellular imaging using emission-tuneable conjugated polymer nanoparticles
Source: RSC Adv. 2019 Nov 20;9(65):37971–6. doi: 10.1039/c9ra07983a (PMC9075778; doi:10.1039/c9ra07983a)
Supplement: RA-009-C9RA07983A-s001 [file RA-009-C9RA07983A-s001.pdf]

Electronic Supplementary Material (ESI) for RSC Advances.
This journal is © The Royal Society of Chemistry 2019

Cellular Imaging using Emission-Tuneable Conjugated Polymer Nanoparticles. Supporting Information.

Struan Bourke, Yurema Teijeiro Gonzalez, Federico Donà, Maryna Panamarova, Klaus Suhling, Ulrike Eggert, Lea Ann Dailey, Peter Zammit and Mark A. Green*

Reagents and Materials

Poly[2-methoxy-5-(2′-ethylhexyloxy)-p-phenylenevinylene] (MEH-PPV) (MW=40,000-70,000), Poly(styrene-co-maleic anhydride), cumene terminated (PSMA) (MW= 1,700), tetrahydrofuran (THF), magnetic iron oxide nanoparticles (10-40 nm, 0.8-1.4% total solid in heptane), pluronic F127 (MW=12.5 kDa avg) and hydrogen peroxide (H_2O_2) (30%) were purchased from Sigma-Aldrich (England, UK). Dulbecco's Modified Eagle Medium (DMEM), fetal bovine serum (FBS), penicillin-streptomycin-glutamine (100X)m ReadyProbes™ Cell Viability Imaging Kit, Blue/Green, ActinGreen ™ ReadyProbes™ Reagent and 4′,6-Diamidine-2′-phenylindole (DAPI) were purchased from Thermofisher Scientific (England, UK). CellTiter-Glo® reagent was purchased from Promega (England, UK). HeLa cells were supplied by Sergi Garcia-Maynes' Lab in the Department of Physics at King's College London, and were used in the Carlton lab and validated by STR profiling from Eurofin MWG[1]. HCEs were a gift from Min S. Chang (Vanderbilt University, Nashville, Tennessee)[2]. HEK cells were supplied by Maryna Panamarova from the Zammit Group in the Randall Division of Cellular and Molecular Biophysics, King's College London.

Instrumentation

Absorption spectra were measured using a Hitachi U-4100 UV-Visible-NIR spectrometer using a 1 cm path length quartz cuvette. Photoluminescence spectra were measured using a Horiba Fluoromax-4 spectrofluorometer. Particle size distributions and zeta potentials were obtained using a Malvern Zetasizer (utilising dynamic light scattering). Transmission electron microscopy images were acquired on a Hitachi 7100 at St George's University of London, with a filament electron source at 100 kV. Image analysis was performed with ImageJ software. The quantum yields (QYs) were measured using the dye comparison method. Photoluminescence quantum yields (PLQYs) were estimated using: quinine sulphate (PLQY = 0.55) in 0.5 M sulfuric acid (H_2O_4)[3]; anthracene (PLQY = 0.36) in cyclohexane[4]; fluoroscein-5-isothiocyanate (FITC, PLQY = 0.79) in ethanol[5]; coumarin 6 (PLQY = 0.78) in ethanol[6]; and rhodamine 6G (PLQY = 0.95) in ethanol[7] as standards.

$$\Phi_{sample} = \Phi_{ref}\left(\frac{I_{sample}}{I_{ref}}\right)\left(\frac{OD_{ref}}{OD_{sample}}\right)\left(\frac{\eta^{2}_{sample}}{\eta^{2}_{ref}}\right)$$

Where the subscripts *sample* and *ref* note sample and reference fluorophore respectively, Φ is the fluorescence QY, I is integrated fluorescence intensity, OD is the optical density at the chosen excitation

wavelength and η is refractive index of the solvent. To avoid self-absorption effects, the absorbance of the samples and the reference fluorophore solutions at the excitation wavelengths were kept below 0.2. Confocal images were acquired with an inverted confocal microscope (Leica TCS SP2) and an internal analogue photomultiplier tube detector, whose detection wavelength range for the fluorescence emission was set to 500-650 nm. The samples were excited with a solid-state CW laser (CNI Laser MBL-III-100mW) at a wavelength of 473 nm and a power of ~5 mW after passing through a neutral density filter wheel. A RSP 500 excitation beam splitter and a 63X 1.2 N.A. water-immersion objective were used to acquire the images. The line scan speed was set to 400 Hz, the image size to 512x512 pixels with a pixel size of 470x470 nm^2 and a pinhole of 2 Airy units. The transmitted light images were taken at the same time as the confocal fluorescence images.

Controlled oxidation of MEH-PPV polymer chains

10mg of MEH-PPV was added to 10 mL of THF (final concentration of 1 mg/mL) and left stirring overnight to ensure MEH-PPV was completely dissolved. At the same time, 10 mL of H_2O_2 (30%) was added to a clear vial. 5 mL of this was added to a vial containing 10 mL of THF. A serial dilution was made, adding 5 mL of the previously diluted H_2O_2 to another 5 mL of THF until a final dilution of 0.03% was made. After a period of time (approximately 1 week), the colour of the solution changed from red to yellow to green to blue, with the photoluminescence and absorption spectra changing also. Typically, a yellow emitting polymer was achieved with 1-2% of H_2O_2, green between 0.1-0.5% H_2O_2, and blue between 0.03% and 0.3% H_2O_2. The emission remained consistent over a year period.

Encapsulation of oxidised MEH-PPV loaded into PSMA

The method was adapted from [8]. 1.5 mL of each oxidised MEH-PPV solutions (0.05 mg/mL) were added to 1.5 mL of THF containing 0.03 mg of PSMA. The solution was sonicated in a 35 kHz ultrasound bath at 7–9 °C, in 30 second bursts for 5 minutes to ensure all polymers were completely dissolved. The solution was then injected into 5 mL deionised water and sonicated for 10 minutes. The solution was then stirred continuously at 400 rpm, at room temperature, for 24 hours to evaporate off THF. Loss of water was compensated by readjustment to 5 mL. The nano-suspension (10 µg/mL of MEH-PPV or total solid of 0.015 mg/mL) was subsequently filtered through a 0.2 µm cellulose acetate Gilson syringe filter. The filtrate was stored at room temperature.

Encapsulation of oxidised MEH-PPV loaded into PSMA with superparamagnetic iron oxide nanoparticles

As above, 1.5 mL of each oxidised MEH-PPV stocks were added to 1.5 mL of THF containing 0.03 mg of PSMA with an additional 10 µL of magnetic nanoparticles (690 mg/mL). The solution was sonicated in a 35 kHz ultrasound bath at 7–9 °C, in 30 second bursts for 5 minutes to ensure all polymers were completely dissolved. The solution was then injected into 5 mL of deionised water and sonicated

for 10 minutes. The solution was then stirred continuously at 400 rpm, at room temperature, for 24 hours to evaporate off THF. Loss of water was compensated by readjustment to 5 mL. The nano-suspension (10 µg/mL of MEH-PPV or total solid of 0.015 mg/mL) was collected by using a magnet and washed. The particles were re-suspended and stored at room temperature.

Encapsulation of oxidised MEH-PPV loaded into F127

The method was adapted from Bourke *et al*, [9]. 1 mL of each oxidised MEH-PPV solutions (0.05 mg/mL) were added to a vial containing 50 mg of F127. The solution was sonicated in a 35 kHz ultrasound bath at 7–9 °C, in 30 second bursts for 5 minutes to ensure all polymers were completely dissolved. The solution was then injected into 9 mL deionised water and sonicated for 10 minutes. The solution was then stirred continuously at 400 rpm, at room temperature, for 24 hours to evaporate off THF. Loss of water was compensated by readjustment to 5 mL. The nano-suspension (10 µg/mL of MEH-PPV or total solid of 10.01 mg/mL) was subsequently filtered through a 0.2 µm cellulose acetate Gilson syringe filter. The filtrate was stored at room temperature.

MEH-PPV-PSMA nanoparticles associated with cells

HeLa and HEK cells were cultured as separate adherent monolayers DMEM supplemented with 10% heat inactivated FBS. HeLa cells were cultured on a sterilised 8 square well microplate, and HEK cells were cultured on a sterilised 96 well microplate. Cell cultures were kept at physiological temperature (~37°C), 5% CO_2 in a humidified incubator. The oxidised MEH-PPV nanoparticle suspension was serially diluted in DMEM to have a polymer concentration of (7.5 µm/mL). 100 µl of the oxidised MEH-PPV nanoparticle suspension was added to 200 µL of the aforementioned media (for the 8 well plate) and 20 µl of the oxidised MEH-PPV nanoparticle suspension was added to 100 µl of the aforementioned media (for the 96 well plate). These were incubated for 24 hours.

HeLa cells were fixed in a 4% formaldehyde solution for 15 minutes, and then washed with phosphate-buffered saline (pH 7.0) six times. All images were acquired on a Leica inverted confocal using a 63X 1.2 N.A. water-immersion objective, with 473 nm excitation. Emission from the oxidised MEH-PPV nanoparticles was collected between 500-650 nm.

For the cytotoxicity study with HEK cells, after the time intervals of 1 hour, 24 hours and 48 hours, 100 µL CellTiter-Glo® reagent was added and cells were left to lyse for 10 minutes before the luminescence was recorded.

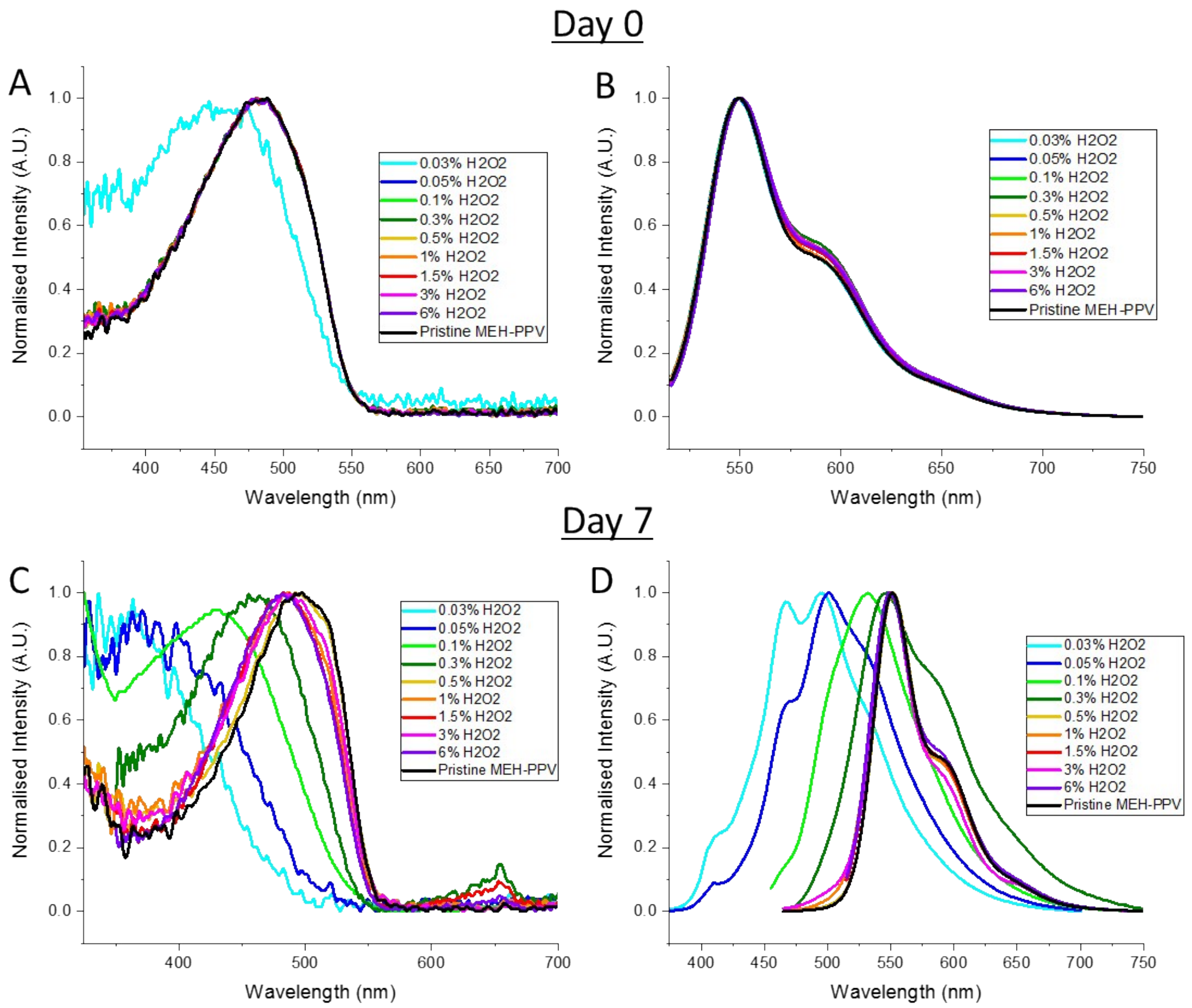

SI figure 1. Normalised absorption and photoluminescence spectra of oxidised MEH-PPV in THF, with changes in emission after seven days. A) and B) indicate that MEH-PPV has no significant blue shifts in emission or absorption at Day 0 (except at 0.03% H_2O_2). C) and D) are the emission and absorption spectra after seven days, where there is a significant blue shift in those solutions with either 0.03%, 0.05%, 0.1% or 0.3% H_2O_2.

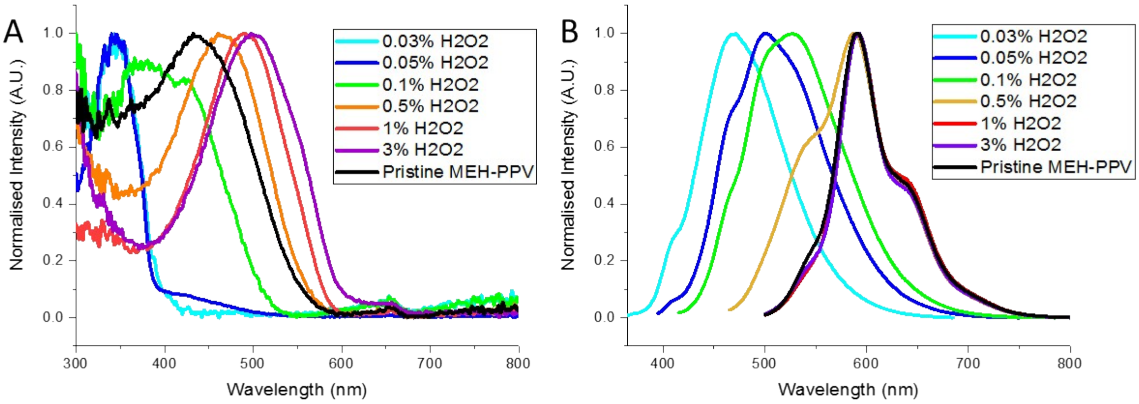

SI figure 2. A) Normalised absorption spectra of oxidised MEH-PPV nanoparticles in F127 micelles, and B) normalised photoluminescence spectra of oxidised MEH-PPV nanoparticles in F127 micelles. Excitation was either at 365 or 500 nm.

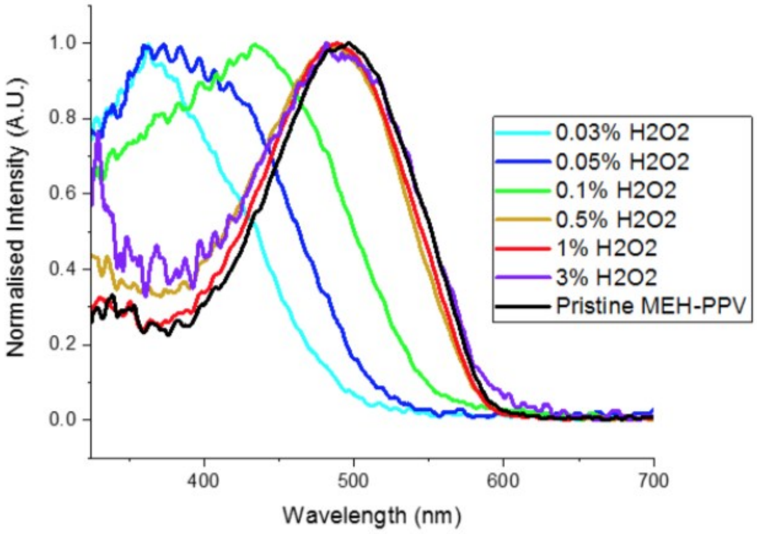

SI figure 3. Normalised absorption spectra of oxidised MEH-PPV nanoparticles capped with PSMA.

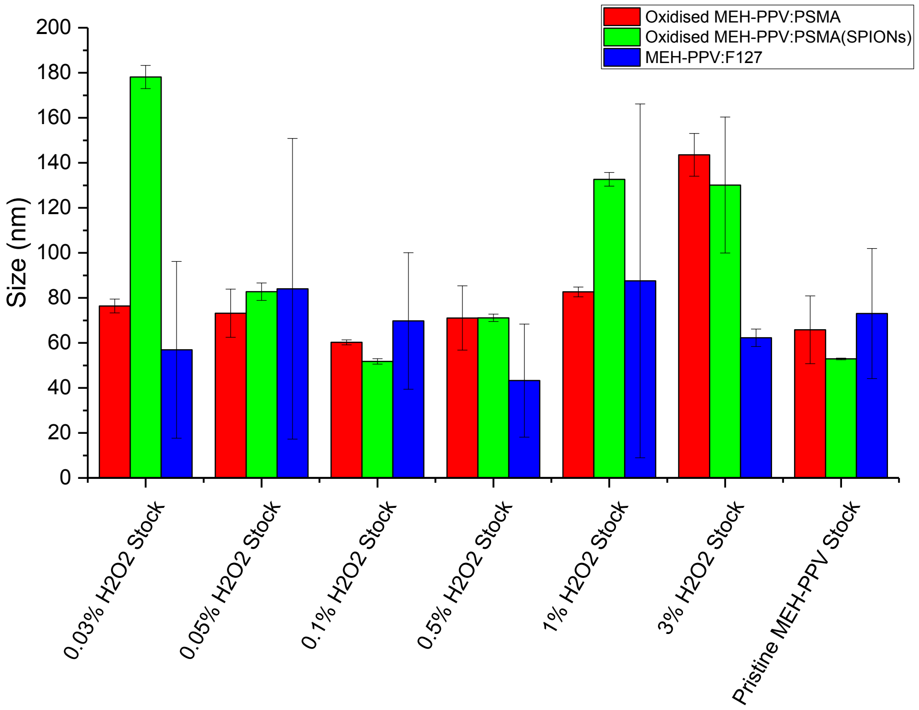

SI figure 4. Graphs of mean diameter/size (nm) of the oxidised MEH-PP NPs. Comparing oxidised MEH-PPV:PSMA without SPIONs (red) and with SPIONs (green), and oxidised MEH-PPV:F127 NPs (blue). (n = 3).

SI Table 1 showing the photoluminescence spectra of MEH-PPV in THF oxidised by different % of H_20_2, and how this changes over 7 days. Abs is absorption, Em is emission and QY is Quantum Yield.

MEH-PPV in THF	Day 0		Day 7		
% of H_2O_2 used	Abs (nm)	Em (nm)	Abs (nm)	Em (nm)	QYs (%)
0.03	450	550	375	450, 475	5
0.05	475	550	375	475	52
0.1	475	550	425	525	71
0.3	475	550	450	530	35
0.5	475	550	475	550	52
1	475	550	475	550	20
3	475	550	475	550	20
6	475	550	475	550	5
Pristine MEH-PPV	475	550	475	550	21

SI Table 2 showing summary of both optical and physical characteristics of the different oxidised MEH-PPV nanoparticles, including their absorption (abs), emission (em), quantum yields (QYs) and Size (nm).

Oxidised MEH-PPV:PSMA NPs (without SPIONs)							
% H_2O_2	0.03	0.05	0.1	0.5	1	3	Pristine MEH-PPV
Abs (nm)	350	375	425	500	500	500	500
Em (nm)	510	525	550	600	600	600	600
QYs (%)	6	26	23	15	6	1	2
Size (nm)	60.2 ± 1.1	52.5 ± 1.2	51.8 ± 1.2	52.9 ± 0.4	80.2 ± 2.2	143.5 ± 9.5	75.5 ± 3.2
PDI	0.11 ± 0.04	0.15 ± 0.06	0.37 ± 0.07	0.28 ± 0.12	0.15 ± 0.03	0.21 ± 0.10	0.32 ± 0.04
Oxidised MEH-PPV:PSMA NPs (with SPIONs)							
% H_2O_2	0.03	0.05	0.1	0.5	1	3	no H_2O_2
Abs (nm)	350	350	375	500	500	500	500
Em (nm)	515	525	550	600	600	600	600
QYs (%)	2	16	15	12	4	1	1
Size (nm)	74.2 ± 9.9	82.7 ± 3.9	88.7 ± 11.3	71.1 ± 1.6	97.9 ± 5.1	176 ± 9.5	87.6 ± 3.8
PDI	0.09 ± 0.06	0.18 ± 0.17	0.44 ± 0.06	0.24 ± 0.08	0.28 ± 0.19	0.41 ± 0.16	0.23 ± 0.05
Oxidised MEH-PPV:F127 NPs							
% H_2O_2	0.03	0.05	0.1	0.5	1	3	no H_2O_2
Abs (nm)	350	350	400	500	525	525	450
Em (nm)	450	475	525	600	600	600	600
QYs (%)	9	35	27	20		1	2
Size (nm)	42.6 ± 3.4	50.3 ± 9.7	70 ± 0.7	92.8 ± 9.8	139.7 ± 38.5	159.6 ± 19.3	62.3 ± 3.9
PDI	0.25 ± 0.05	0.34 ± 0.09	0.34 ± 0.2	0.45 ± 0.2	0.24 ± 0.10	0.43 ± 0.03	0.31 ± 0.051

Reference

(1) Olmos, Y.; Perdrix-Rosell, A.; Carlton, J. G. Membrane Binding by CHMP7 Coordinates ESCRT-III-Dependent Nuclear Envelope Reformation. *Curr. Biol.* **2016**, *26* (19), 2635–2641. https://doi.org/10.1016/j.cub.2016.07.039.

(2) Terry, S. J.; Zihni, C.; Elbediwy, A.; Vitiello, E.; San, I. V. L. C.; Balda, M. S.; Matter, K. Spatially Restricted Activation of RhoA Signalling at Epithelial Junctions by P114RhoGEF Drives Junction Formation and Morphogenesis. *Nat. Cell Biol.* **2011**, *13* (2), 159–166. https://doi.org/10.1038/ncb2156.

(3) Eaton, D. F. Reference Materials for Fluorescence Measurement. *Pure Appl. Chem.* **1988**, *60* (7). https://doi.org/10.1351/pac198860071107.

(4) Berlman, I. B. *Handbook of Fluorescence Spectra of Aromatic Molecules*; Elsevier, 1971. https://doi.org/10.1016/B978-0-12-092656-5.50006-X.

(5) Kellogg, R. E.; Bennett, R. G. Radiationless Intermolecular Energy Transfer. III. Determination of Phosphorescence Efficiencies. *J. Chem. Phys.* **1964**, *41* (10), 3042–3045. https://doi.org/10.1063/1.1725672.

(6) Reynolds, G. A.; Drexhage, K. H. New Coumarin Dyes with Rigidized Structure for Flashlamp-Pumped Dye Lasers. *Opt. Commun.* **1975**, *13* (3), 222–225. https://doi.org/10.1016/0030-4018(75)90085-1.

(7) Kubin, R. F.; Fletcher, A. N. Fluorescence Quantum Yields of Some Rhodamine Dyes. *J. Lumin.* **1982**, *27* (4), 455–462. https://doi.org/10.1016/0022-2313(82)90045-X.

(8) Wu, C.; Hansen, S. J.; Hou, Q.; Yu, J.; Zeigler, M.; Jin, Y.; Burnham, D. R.; McNeill, J. D.; Olson, J. M.; Chiu, D. T. Design of Highly Emissive Polymer Dot Bioconjugates for in Vivo Tumor Targeting. *Angew. Chemie - Int. Ed.* **2011**, *50* (15), 3430–3434. https://doi.org/10.1002/anie.201007461.

(9) Bourke, S.; Urbano, L.; Olona, A.; Valderrama, F.; Ann Dailey, L.; Green, M. A.; Ann, L.; Mark, A. Silica Passivated Conjugated Polymer Nanoparticles for Biological Imaging Applications. *Prog. Biomed. Opt. Imaging - Proc. SPIE* **2017**, *10079*, 1–13. https://doi.org/10.1117/12.2252035.
